# Henry gas solubility optimization double machine learning classifier for neurosurgical patients

**DOI:** 10.1371/journal.pone.0285455

**Published:** 2023-05-11

**Authors:** Diana T. Mosa, Amena Mahmoud, John Zaki, Shaymaa E. Sorour, Shaker El-Sappagh, Tamer Abuhmed

**Affiliations:** 1 Department of Information Systems, Faculty of Computers and Information, Kafrelsheikh University, Kafr El-Shaikh, Egypt; 2 Department of Computer Sciences, Faculty of Computers and Information, Kafrelsheikh University, Kafr El-Shaikh, Egypt; 3 Department of Computer and Systems, Faculty of Engineering, Mansoura University, Mansoura, Egypt; 4 Preparation- Computer Science and Education, Faculty of Specific Education, Kafrelsheikh University, Kafr El-Shaikh, Egypt; 5 Faculty of Computer Science and Engineering, Galala University, Suez, Egypt; 6 Faculty of Computers & Artificial Intelligence, Benha University, Banha, Egypt; 7 College of computing and informatics, Sungkyunkwan University, Seoul, Republic of Korea; Jeonbuk National University, KOREA, REPUBLIC OF

## Abstract

This study aims to predict head trauma outcome for Neurosurgical patients in children, adults, and elderly people. As Machine Learning (ML) algorithms are helpful in healthcare field, a comparative study of various ML techniques is developed. Several algorithms are utilized such as k-nearest neighbor, Random Forest (RF), C4.5, Artificial Neural Network, and Support Vector Machine (SVM). Their performance is assessed using anonymous patients’ data. Then, a proposed double classifier based on Henry Gas Solubility Optimization (HGSO) is developed with Aquila optimizer (AQO). It is implemented for feature selection to classify patients’ outcome status into four states. Those are mortality, morbidity, improved, or the same. The double classifiers are evaluated via various performance metrics including recall, precision, F-measure, accuracy, and sensitivity. Another contribution of this research is the original use of hybrid technique based on RF-SVM and HGSO to predict patient outcome status with high accuracy. It determines outcome status relationship with age and mode of trauma. The algorithm is tested on more than 1000 anonymous patients’ data taken from a Neurosurgical unit of Mansoura International Hospital, Egypt. Experimental results show that the proposed method has the highest accuracy of 99.2% (with population size = 30) compared with other classifiers.

## 1 Introduction

Traumatic brain injury (TBI) is a significant health challenge that causes death around the world as it contributes to almost one third of all trauma-related mortalities [1, 2]. Surviving patients often suffer from long-term physical and cognitive shortages, with devastating consequences for the patients and their families [3]. Studies found that head injury (HI) is a common reason for being admitted to the emergency department with the highest rates of TBI–related visits occurring among children and senior citizens [2]. It may damage a nerve tissue and cause disorder of consciousness, vision problems, headaches, and sleep issues [4]. Reports show that nearly 5.3 million people in USA alone have functional disabilities and over 10 million people worldwide were admitted to hospitals or deceased [3, 5] as a result of falling, road accidents or sports [6]. For instance, Sports and military people may have been exposed to recurrent HI situations that cause risk of Alzheimer or multiple sclerosis diseases [7–11].

Traumatic brain injury assessment has a number of complications and difficulties. Several researchers exerted too much effort in attempt to classify TBI. Recently, Machine Learning (ML) is gaining popularity in the medical field. It helps to understand patients’ conditions and develop prediction models for better treatment [12, 13]. ML algorithms can process a vast number of attributes in datasets and offer better classification accuracy [14, 15]. Machine learning can be used to develop prediction models for medical datasets. With the help of feature selection techniques, it can reduce dimensionality in the dataset by determining the most important attributes [12, 16].

The main aim of this paper is to predict the status outcome of Neurosurgical patients with high accuracy and employ necessary features. In this regard, known machine learning algorithms were used to detect neurosurgical patient outcome. Moreover, Henry Gas Solubility Optimization (HGSO) was employed with Aquila Optimizer (AQO). Comparative study of different ML techniques such as K-NN, Random Forest (RF), C4.5, ANN, and SVM classifiers was carried out. The comparative study experimented “with and without the optimized HGSO”.

Later, a new method for patients’ outcome status prediction was proposed. Its accuracy, F-measure and sensitivity were calculated. Finally, it was found that the hybrid prediction model based on SVM & RF with the optimized HGSO algorithm has the highest accuracy.

The main contributions of this research are as follows:

1. A series of experiments are conducted over the mentioned neurosurgical dataset regarding the problem of feature selection and classification using various machine learning algorithms with HGSO to evaluate the efficiency of the proposed HGSO. The results of these experiments may serve as important inputs for further research.
2. HGSO is able to avoid local optima and maintain the balance between the exploration and exploitation phases compared to other competitive metaheuristic algorithms.

The remainder of this paper is structured as follows: The next section discusses the recent related research. Section three discusses the materials and methods. Section four talks about the experimental design of the study. In section five, the results and the comparative analysis of the different classifiers. The conclusion is given in section seven.

## 2 Literature review

Hale et al. [17] used ANN to accurately predict six months outcomes in pediatric patients with TBI by comparing their ANN analysis to both conventional statistical models and predictive models based on CT classification schemes. Furthermore, they discuss the evolution of predicting the outcome of patients with TBI and delineate the ANN approach for medical diagnosis, prognosis, and management [18].

Tunthanathip et al. [19] predicted surgical site infection (SSI) after neurosurgical operations using decision trees (DT), Naive Bayes (NB) with Laplace correction, KNN, and ANNs. They tested the algorithms on 1471 patients who had undergone neurosurgical operations at tertiary care hospitals between 2010 and 2017. NB algorithm is highlighted as an accurate ML method with 63% sensitivity at, 87% specificity, 29% positive predictive value, 96% negative predictive, and 76% area under the receiver operating characteristic curve (AUC).

Raju et al. [20] used ML algorithms to train and optimize outcomes of neurological signals by monitoring vital signs or other electrophysiological indicators (motor evoked potentials) to provide feedback and improving modulation control. Scheer et al. [21] predicted major complications in adult spinal deformity surgery by applying multiple bootstrapped decision trees on available patients. The model achieved an AUC of 0.89 and accuracy of 87% at internal validation. However, no sensitivity or specificity were reported [22].

In a recent Swiss study exploring the attitudes of neurosurgeons toward ML, Staartjes et al. [22] found that 29% of the 362 participants surveyed were already implementing ML into their practice and a further 31% using it for research purposes. ML assisted neurosurgeons through improving the preoperative and intraoperative decision-making process, enhancing objectivity in the diagnosis, and anticipating complications [23, 24].

Abujaber et al. [25] used logistic regression (LR) and ANN to predict the in hospital mortality for 785 adult patients on mechanical ventilation following moderate to severe TBI. They used their demographic characteristics, injuries and CT findings as predictors. The LR model achieved 87% accuracy and 90.5% area under the receiving-operating characteristic curve (AUROC) while the ANN achieved accuracy and AUROC of 80.9% and 87.5% respectively.

Mofatteh [26] article reviewed some studies in ML for multiple neurosurgical domains. Some of the reviewed papers used ML to classify lumbar disk degeneration using MRI scans from healthy to severely abnormal disks. Other papers utilized ML to cluster patients suffering from osteoporotic vertebral fracture based on their pain progression. Additionally, ML helped diagnosing pediatric posterior fossa tumors by categorizing them into the primitive neuroectodermal tumor, astrocytoma, or ependymal with 72% accuracy compared to 73% accuracy of neuroradiologists. Further studies showed that ANN predicted the glioma according to the World Health Organization grade better than radiologists. Beyond tumor diagnosis, ML outperformed physicians with 82.2% to 62.2% accuracy in predicting the presence of abnormal features in CT scans of pediatric TBI patients [26].

Other studies tried to apply ML methods to neuroimaging data to assist with stroke diagnosis. Used SVM in resting-state functional MRI data, SVM can correctly classify patients with stroke with 87.6% accuracy. Kamnitsas et al. [27] tried three-dimensional CNN for lesion segmentation in multimodel brain MRI. They also used fully connected conditional random field model for final postprocessing of the CNN’s soft segmentation maps. Rondina et al. [28] analyzed stroke anatomical MRI images using Gaussian process regression, and found that the patterns of voxels performed better than lesion load per region as the predicting features. ML methods have also been applied to analyze CT scans from patients with stroke.

Thornhill et al. [29] used linear discriminant analysis, artificial neural network and SVM to classify lesion after stroke and carotid plaque on the CT imaging, the accuracy for each method varies between 65.2% and 76.4%. Asadi et al. [30] analyzed 107 patients of acute anterior or posterior circulation stroke via ANN and SVM. The research obtained prediction accuracy above 70%. They also used ML techniques to identify factors influencing outcome in brain arteriovenous malformation treated with endovascular embolization with 97.5% accuracy. Birkner et al. [31] used an optimal algorithm to predict 30-day mortality and obtained more accurate prediction than existing methods. Similarly, King et al. [32] used SVM to predict stroke mortality at discharge. In addition, they proposed the use of the synthetic minority oversampling technique to reduce the stroke outcome prediction bias caused by between-class imbalance among multiple data sets [5].

In 2017, Subasi et al. [33] proposed an algorithm to detect epileptic seizures in Electroencephalography (EEG) using SVMs and Genetic Algorithms which proved an accuracy of 99.38% on the EEG dataset. However, Avcu et al. [34] used Convolutional Neural Network (CNN) to detect seizures using only two channels with an accuracy of 93.3% [35].

Prashanth et al. [14] presented an ML system that can accurately predict Parkinson’s disease with an accuracy of 96.40%. Rastegar et al. [36] predicted disease progression using serum cytokines from one time point (baseline); then, after one year, to predict the outcome for two years [35].

Buchlak et al. [23] article compared the top three most frequently applied ML algorithms in neurosurgery namely LR, ANN, and SVM. They found that the accuracy and specificity of ANN, LR, and SVM differ significantly where ANN algorithm demonstrated higher accuracy than LR while SVM demonstrated higher specificity than LR. Nevertheless, they found no significant difference in AUC and sensitivity among ANN, LR, and SVM [23].

Vivaldi et al. [37] suggested that EEG data-driven ML using SVM and KNN models can be a useful tool to distinguish between TBI and normal patients. The results showed 94% accuracy and 94% sensitivity in cross validation while it showed 76% accuracy and 80% sensitivity in independent validation.

Brossard et al. [38] article focused on the classification and the segmentation of lesions. They used manual and automated analysis of CT scans. The study developed an automated determination of traumatic brain lesions and medical-decision process using supervised learning and CT scans for patients with TBI. The method enhanced the quantitative analysis of CT images and offered a new perspectives in clinical care of TBI.

Noor and Ibrahim [39] reviewed 40 different studies that evaluated ML algorithms using quantitative EEG (qEEG) predictors that predict outcome in patients with moderate to severe TBI. The most common ML technique used was LR with the highest accuracy. However, the algorithms varied depending on the type and number of qEEG predictors selected in each model. The qEEG variability for the relative and absolute band powers were the most common qEEG predictors included in the models followed by total EEG power of all frequency bands, EEG-reactivity, and coherence. Model performance was measured by AUROC rather than by accuracy rate. Various ML models demonstrated great potential especially using qEEG predictors.

Radabaug et al. [40] tried to overcome a lack of translation from laboratory research to clinical application using SL. They built a clinically-relevant evaluation metric that treats a memory retention task (i.e. probe trial) as the class label. They used univariate statistical analysis on an Operation Brain Trauma Therapy dataset. The prediction accuracy was 67% by NB on the borderline elimination dataset.

Thanjavur et al. [41] introduced a deep learning long-short term memory based recurrent neural network. The algorithm was able to distinguish between non-concussed and acute post-concussed adolescent athletes using only short (i.e. 90s long) samples of resting state EEG data as input. The network was trained and validated using data from 27 male, adolescent athletes with sports related concussion and benchmarked against 35 non-concussed adolescent athletes. During rigorous testing, the classifier consistently identified concussions with an accuracy greater than 90% and achieved an ensemble median Area Under the Receiver Operating Characteristic Curve (ROC/AUC) equal to 0.971 [36].

Siyar et al. [42] outlined the first application of ML to distinguish “skilled” and “novice” psychomotor performance during virtual reality brain tumor resection tasks. The tasks remove a series of virtual brain tumors without causing injury to the surrounding tissue. The application fed features to KNN, Parzen Window, SVM, and Fuzzy KNN. Additionally, sets of 5 to 30 selected features were provided to the classifiers. A working point of 15 premium features resulted in accuracy values as high as 90% using SVM [42].

In a recent study by Vishwanath et al. [43], various ML algorithms were used. Namely,rule-based algorithms of decision trees, random forest, neural network, SVM, KNN, and CNN to classify TBI data obtained from the proposed mouse model. The use of CNN for sleep-wake data yielded the highest accuracy indicating a promising method for accurate identification of the relevant brain-based biomarkers in TBI. The results obtained for rule-based methods and CNN are comparable. Overall, the highest classification accuracy of 92.03% was obtained by CNN when the entire EEG signal (both wake and sleep stages) was used.

Susheela and Ajit [44] introduced an improved Henry gas solubility optimization in which the selected features were the input to the classifiers. They were used to identify histopathological images. There were 23 benchmark functions employed for the performance evaluation of the enhanced Henry gas solubility optimization. ICIAR’s grand challenge dataset and the breast cancer cell dataset were used to test the suggested feature selection approach. Using this feature selection strategy, the two datasets were reduced by 60% on average.

To improve classification accuracy, Nabil Neggaz et al. [45] proposed an approach for dimensionality reduction based on the Henry gas solubility optimization (HGSO) method for selecting significant features. The suggested technique employs the expert systems of k-nearest neighbour (k-NN) and support vector machine (SVM) to assess the chosen set of features, and it is compared to well-known meta-heuristic algorithms. Overall, the empirical analysis suggests that the proposed approach is significantly effective by producing 100% accuracy on classification problems with more than 11,000 features.

Thus, ML-based algorithms are promising in TBI to predict patients’ outcomes more accurately than conventional analysis. This is due to their high diagnostic accuracy, analysis, and detection. Therefore, ML opens the door to prospective research areas in brain tumors and therapeutics that were never otherwise possible [46, 47].

ML can be used to develop prediction models for medical datasets. With the help of feature selection techniques, it can reduce dimensionality in the dataset by determining the most important attributes [15, 48].

## 3 Materials and methods

This section shows a discussion for different ML techniques, which is known as data-driven AI [33, 49]. Moreover, the mathematical formulation of the used feature selection method to reduce the number of features is introduced.

### 3.1 Data collection

In this research, Table 1 describes the training data that was collected from a Neurosurgical unit at Mansoura International Hospital in Egypt. There are 1160 patients. The medical attributes were retrieved while the identity of the patients was anonymized.

**Table 1:** Patient features and their domain.

Domain	Features
Personal Data	Name, Age, Gender,
	Address, Phone, Special Habits
History	Type of head injury,
	Mode of Trauma
	Previous Admission
	Comorbidity
	Previous Operation
Clinical Data	Pulse/min.,
	Temperature,
	Blood Pressure (mmHg)
	Respiration/min.
	Admission GCS /15: EO VR MR
	Neurological deficit Quadriparesis
	Pupils
	Cranial Nerves
Associated injuries	Spinal, Abdominal
	Chest, Long bones
Laboratory Data	Blood Picture
	Electrolytes
	Blood Sugar
	Renal
	Hepatic
	Others
Radiology	Skull Fractures
	DAI
	Concussion
	Contusions
	EDH Location / Volume
	SDH Acute / Chronic
	SAH (Subarachnoid hemorrhage)
	ICH Location / Volume
	Pneumocephalus Location/amount
Degree of Head Injury	-
Intervention Medical Surgical	Measure the improvement—for 2 weeks
GOS Evaluation at discharge	after 2 weeks
Outcome	Mortality– Morbidity—The same—Improved

### 3.2 Data preprocessing

Generally, real data is incomplete, inconsistent and noisy. Therefore, it is crucial to perform preprocessing activities to prepare the data. The preprocessing includes data cleaning, transformation, extraction and coding of attributes to perform normalization. The dataset contains data records of 40 attributes for various patients. Personal data such as name, address, and phone number attributes were removed by the hospital prior to providing the data for research resulting in a total of 37 medical attributes.

### 3.3 Methods

The proposal for this work was submitted to the research and ethical committee at The General Organization for Teaching Hospitals and Institutes (GOTHI: https://gothi.gov.eg/). It does not include any details regarding the participants’ consent as the written consent was obtained by the hospital collecting the data. Anonymized data was provided to the authors. The research does not include any minors. It earned the IRB approval number HS000106.

#### 3.3.1 Aquila Optimizer (AQO)

Aquila Optimizer [50] is a revolutionary population-based optimization approach that is based on the Aquila’s behavior while it hunts. Therefore, it is possible to express the optimization processes of the proposed AQO algorithm in four ways: high soar with vertical stoop; contour flight with short glide attack; low flight with slow descent assault; and swooping by walk and capture prey, all of which may be applied to the search space.

To begin the process of AQO, the population of potential solutions (X) is created stochastically between the upper bound (UB) and lower bound (LB) of the given issue. The optimization rule is derived from this population. During each iteration, the best-obtained solution is found to be an approximate optimum solution for the problem at hand.

The AQO algorithm can transfer from exploration steps to exploitation steps using different behaviors based on the condition: $if\;t\leq \frac{2}{3}T$ the exploration steps will be executed. Otherwise, the exploitation steps will be executed. As a mathematical optimization paradigm, Aquila’s behavior is characterized by discovering the optimum solution given a set of specified restrictions. AQO’s mathematical model is presented in the following manner.

*Generation of initial population*. In order to demonstrate the effectiveness of the provided AQO, the tested benchmark data is first divided into a training set consisting of 80% of the data and a testing set consisting of 20% of the data. The following Equation creates the initial population *X*, which is made up of *N* solutions:

$$\begin{array}{c} X_{i}=LB+rand\left(1,D\right)\times (UB-LB) \end{array}$$ (1)

Where *D* is the number of features. *rand*(1, *D*) is a random D-dimensional vector. The search space’s perimeters are symbolized by *LB* and *UB*.

*Updating population*. The following equation Eq 2 transforms *X*_*i*_, *i* = 1, 2, …, *N* into its Boolean value *BX*_*i*_ at the beginning of this step.

$$\begin{array}{c} BX_{i}=\{\begin{array}{cc} 1 & if\;X_{ij}>0.5\\ 0 & otherwise \end{array} \end{array}$$ (2)

It is possible to limit feature selection based on Eq 2 result by discarding the useless features that have zero values in *BX*_*i*_. Once the fitness value is determined, it may be calculated using Eq 3 below:

$$\begin{array}{c} Fit_{i}=\lambda \times \gamma_{i}+\left(1-\lambda \right)\times \frac{|BX_{i}|}{D} \end{array}$$ (3)

This is followed by a determination of the best fit and its associated best agent *X*_*b*_. Then add the AQO operators to the present agents.

*Terminal criteria*. At this step, the stopping criteria is evaluated. If it is not fulfilled, the update stage is repeated. Otherwise, the learning process is finished, and *X*_*b*_ is used as the result to reduce the testing set.

*Validation stage*. It is necessary to minimize the testing set characteristics in order to assess how well AQO performs as a feature selection strategy. Finally, several performance indicators are used to evaluate the classification process quality based on the reduced characteristics.

#### 3.3.2 Henry Gas Solubility Optimization (HGSO)

**Fig 1:**
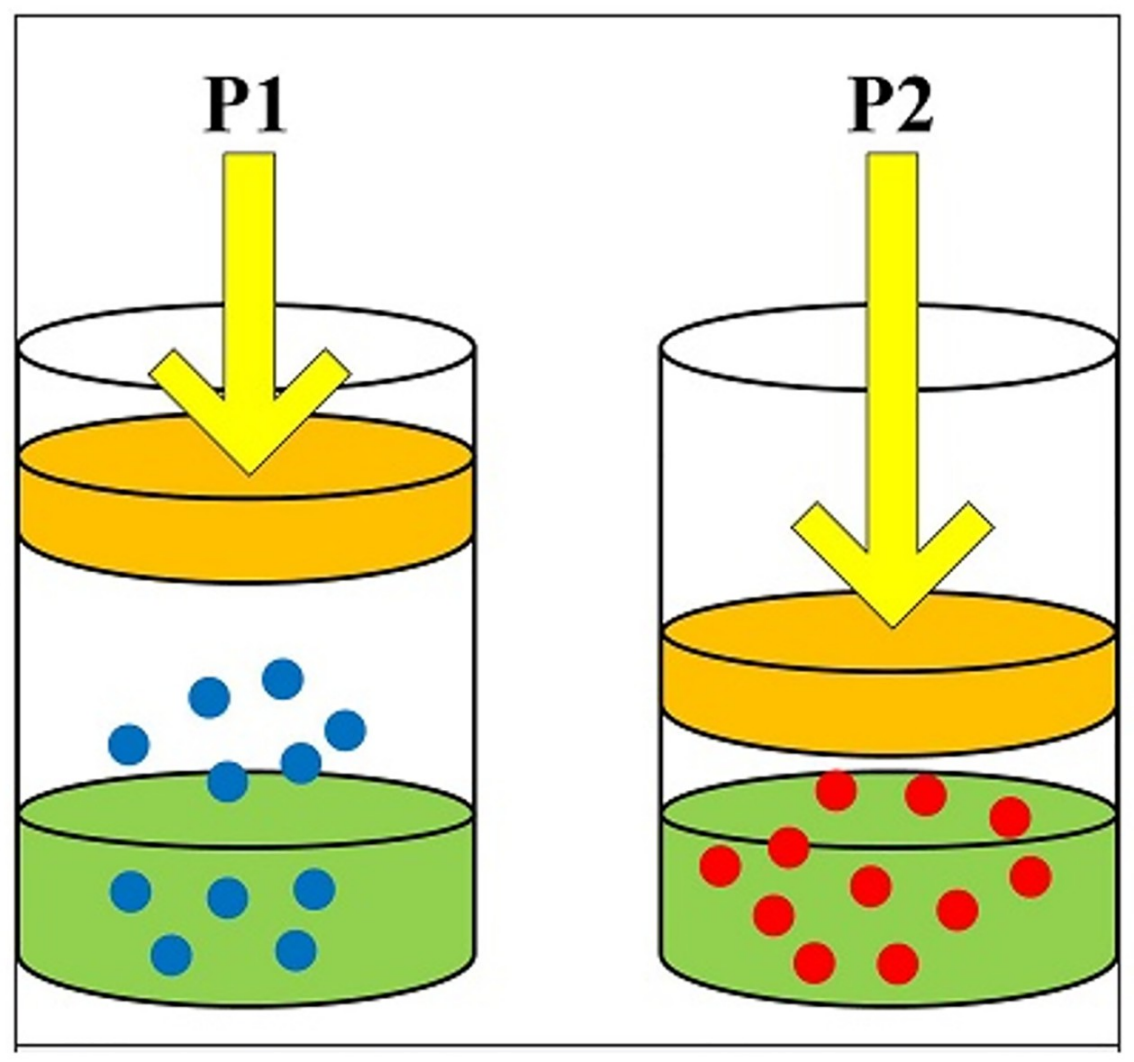
Henry gas solubility bases.

In 2019, Hashim et al. [51] suggested a metaheuristic algorithm derived from William Henry’s law of physics which describes gas particles in a liquid with partial pressure namely HGSO. Henry’s law depends on the dissolved gas amount, liquid type and volume at a specific temperature. For instance, this phenomenon exists on carbonized beverages cans. (Fig 1) shows huddling behavior of gas particles with 2 different pressures [51–53]. As shown in (Fig 1), when the pressure rises, extra gas particles dissolve till reaching the equilibrium again.

Henry suggested the following equation as the gas solubility *S*_*g*_ proportions directly with the gas partial pressure *P*_*g*_:

$$\begin{array}{c} S_{g}=H\times P_{g} \end{array}$$ (4)

where *H* is Henry’s constant.

Henry’s law constants affected by the temperature changes, Van’t Hoff equation describes it:

$$\begin{array}{c} \frac{d\;\text{ln}\;H}{d(1/T)}=\frac{-{▽}_{sol}E}{R} \end{array}$$ (5)

where (▽_*sol*_*E*) is the dissolution enthalpy, *R* is the gas constant, and the temperature *T* depends on *H*.

After integrating Eq 4, it becomes:

$$\begin{array}{c} H\left(T\right)=e^{\left(B/T\right)}\times A \end{array}$$ (6)

Where A and B are parameters that depends on *H* and *T* = 298.15*K*.

$$\begin{array}{c} H\left(T\right)=H^{\theta }\times exp(\frac{-{▽}_{sol}E}{R}\times \left(\frac{1}{T}-\frac{1}{T^{\theta }}\right)) \end{array}$$ (7)

when the dissolution enthalpy ▽_*sol*_*E* is a constant, the Van’t Hoff equation is valid. therefore, Eq (4) becomes:

$$\begin{array}{c} H\left(T\right)=exp(-C\times (\frac{1}{T}-\frac{1}{T^{\theta }}))\times H^{\theta } \end{array}$$ (8)

Equations Eq 4 through Eq 8 are used to calculate the solubility in liquids for low solubility gas.

#### 3.3.3 Mathematical model of HGSO algorithm

The algorithm used in this research mimics the gas behavior in equilibrium of exploitation, exploration, and escapes the local optima of the HGSO inspiration in the search space.

**Initialization**: The gases number N and their positions are set according to Eq 9.

$$\begin{array}{c} X_{i}\left(t+1\right)=X_{min}+r\times \left(X_{max}-X_{min}\right) \end{array}$$ (9)

where *X*_*i*_ is the *i*^*th*^ gas position in population *N*, *t* is the time of iteration, *r* is a randomly chosen number between 0 and 1. The problem bounds are *X*_*min*_ and *X*_*max*_.

The values of the following terms are set according to Eq 10 where *i* is the gas number, *H*_*j*_(*t*) is Henry’s constant of type *j*. *P*_*i*,*j*_ is the partial gas pressure *i* in the *j*’s cluster, ▽_*sol*_*E*/*R* is a number named *C*_*j*_.

$$\begin{array}{ccc} H_{i}\left(t\right) & = & l_{1}\times rand\left(0,1\right)\\ P_{i,j} & = & l_{2}\times rand\left(0,1\right)\\ C_{j} & = & l_{3}\times rand\left(0,1\right) \end{array}$$ (10)

where the constants *l*_1_ = 5*E* − 02, *l*_2_ = 100, *l*_3_ = 1*E* − 02.

**Clustering**: The population gases are distributed to equivalent clusters with similar gases according to the gas type’s number and the same value of *H*_*j*_.

**Assessment**: To detect the best gas cluster which helps to reach the maximum equilibrium state than other clusters in the same type, each gas cluster *j* is evaluated and the clusters are ordered to find the optimal cluster for this swarm.

**Update Henry’s Coeffiecient**: The update of the coefficient happens according to the following equation where *H*_*j*_ is Henry’s coefficient for cluster *j*, *T* is the temperature, *T*^*θ*^ = 298.15 and *iter* is the total number of iterations.

$$\begin{array}{c} H_{j}\left(t+1\right)=H_{j}\left(t\right)\times exp(-C_{j}\times \left(\frac{1}{T(t)}-\frac{1}{T^{\theta }}\right))\;,T\left(t\right)=exp\left(\frac{-t}{iter}\right) \end{array}$$ (11)

**Update the solubility of Gas**:

$$\begin{array}{c} S_{i,j}\left(t\right)=K\times H_{j}\left(t+1\right)\times P_{i,j}\left(t\right) \end{array}$$ (12)

where *S*_*i*,*j*_ is the *i*^*th*^ gas solubility in the *j*^*th*^ cluster, *P*_*i*,*j*_ is the partial pressure on gas *i* in cluster *j* and *K* is a constant.

**Change the position**: This happens according to the solubility from the objective function:

$$\begin{array}{ccc} X_{i,j}\left(t+1\right) & = & X_{i,j}\left(t\right)+F\times r\times \gamma (X_{i,best}\left(t\right)-X_{i,j}\left(t\right))\\ & + & F\times r\times \alpha (S_{i,j}\left(t\right)X_{best}\left(t\right)-X_{i,j}\left(t\right)) \end{array}$$ (13)

$$\begin{array}{c} \gamma =\beta \times exp(-\frac{F_{best}\left(t\right)+\epsilon }{F_{i,j}\left(t\right)+\epsilon }) \end{array}$$ (14)

where *X*_*i*,*j*_ is the position of gas *i* in cluster *j*, *r* is a random constant and *t* is the iteration time. The best gas *i* in cluster *j* is denoted by *X*_*i*,*best*_, and *X*_*best*_ is the best gas in the swarm. The parameters *X*_*i*,*best*_ and *X*_*best*_ are used to achieve the balance between the exploration and exploitation. Moreover, *Y* is the interaction ability of gas *i* in cluster *j* and other gases in the same cluster, *α* is the other gases effect on gas *i* in cluster *j* = 1 and *β* is a constant. *F*_*i*,*j*_ is the fitness of gas *i* in cluster *j*, while *F*_*best*_ is the fitness of the best gas in the system. *F* denotes the direction flag for the search agent that offers diversity = ±.

**Escape from the local optima**: The agents are ordered and the worst agents number *Nw* is selected according to the following Equation:

$$\begin{array}{c} N_{w}=N\times (rand\left(c_{2}-c_{1}\right)+c_{1}),\;c_{1}=0.1\;and\;c_{2}=0.2 \end{array}$$ (15)

where *N* is the search agent number.

**Modify the worst agent position**:

$$\begin{array}{c} G_{i,j}=G_{min(i,j)}+r\times \left(G_{max(i,j)}-G_{min(i,j)}\right) \end{array}$$ (16)

where *G*_*i*,*j*_ is the position of gas *i* in cluster *j*, *G*_*min*_ and *G*_*max*_ are the problem boundaries while *r* is a random number.

**Algorithm 1**: HGSO algorithm pseudo code

**Begin**: *X*_*i*_(1, 2, 3…*N*, gas types number *i*, *H*_*j*_, *P*_*i*,*j*_, *C*_*j*_, *l*_1_, *l*_2_, and *l*_3_

**Compute**: Partition the agents of population into clusters of gas types which have the same value of Henry’s constant *H*_*j*_.

**Compute**: Assess each cluster *j*.

**Compute**: Find the value of best gas *X*_*i*,*best*_ for all clusters, also the value of best search agent *X*_*best*_.

**repeat**

 **for** each *X*_*best*_ **do**

  update the values for all *X*_*best*_’s using Eq 13

 **end**

 **Modify**: *H*_*j*_ for each type of gas using Eq 11.

 **Modify**: solubility value for each gas using Eq 12.

 **Compute**: Order and determine the worst agents number using Eq 15

 **Modify**: the value of the worst agents position using Eq 16.

 **Modify**: *X*_*i*,*best*_ and *X*_*best*_.

**until** *t* < *maximum iterations number*;

**Compute**: *t* = *t* + 1

**return** *X*_*best*_

The complexity of HGSO algorithm is of order: *O*(*tnd*) × *O*(*obj*), where *t* is the maximum number of iterations, *n* is the number of solutions, *d* is the number of variable, and *obj* is the objective function.

The key control parameter of HGSO is the balance between exploration (the increased mean value of distance via population dimension) and exploitation (the reduced mean value) phases. To determine the dimension-wise variety through search iterations, the following equation is used:

$$\begin{array}{c} \frac{1}{Div_{j}}=\frac{1}{N}\sum_{i=1}^{N}median\left(x^{j}\right)-x_{i}^{j},\;Div^{t}=\frac{1}{N}\sum_{j=1}^{D}Div_{j} \end{array}$$ (17)

where, $X_{i}^{j}$ is the *j*^*th*^ dimension of *i*^*th*^ population individual, median *x*_*j*_ is the median value of *j*^*th*^ dimension of the population with size *N*, *Div*_*i*_ is the mean variety measure for dimension *j*, and *Div*^*t*^ is the average of the *D* dimensions for iteration *t* where *t* = 1, 2, 3, …, *iter*. When the population diversity is determined for maximum iterations, the search processes *iter* calculates the percentage of exploration and exploitation in the the search process as shown in the following equations:

$$\begin{array}{c} Exploration\%=\frac{Div^{t}}{Div_{max}}\times 100 \end{array}$$ (18)

$$\begin{array}{c} Exploitation\%=\frac{|Div^{t}-Div_{max}|}{Div_{max}}\times 100 \end{array}$$ (19)

where *Div*_*max*_ is the maximum variety of iterations *t*. Finally, HGSO can achieve a balance among the factors.

## 4 Experimental setup

### 4.1 Design

**Fig 2:**
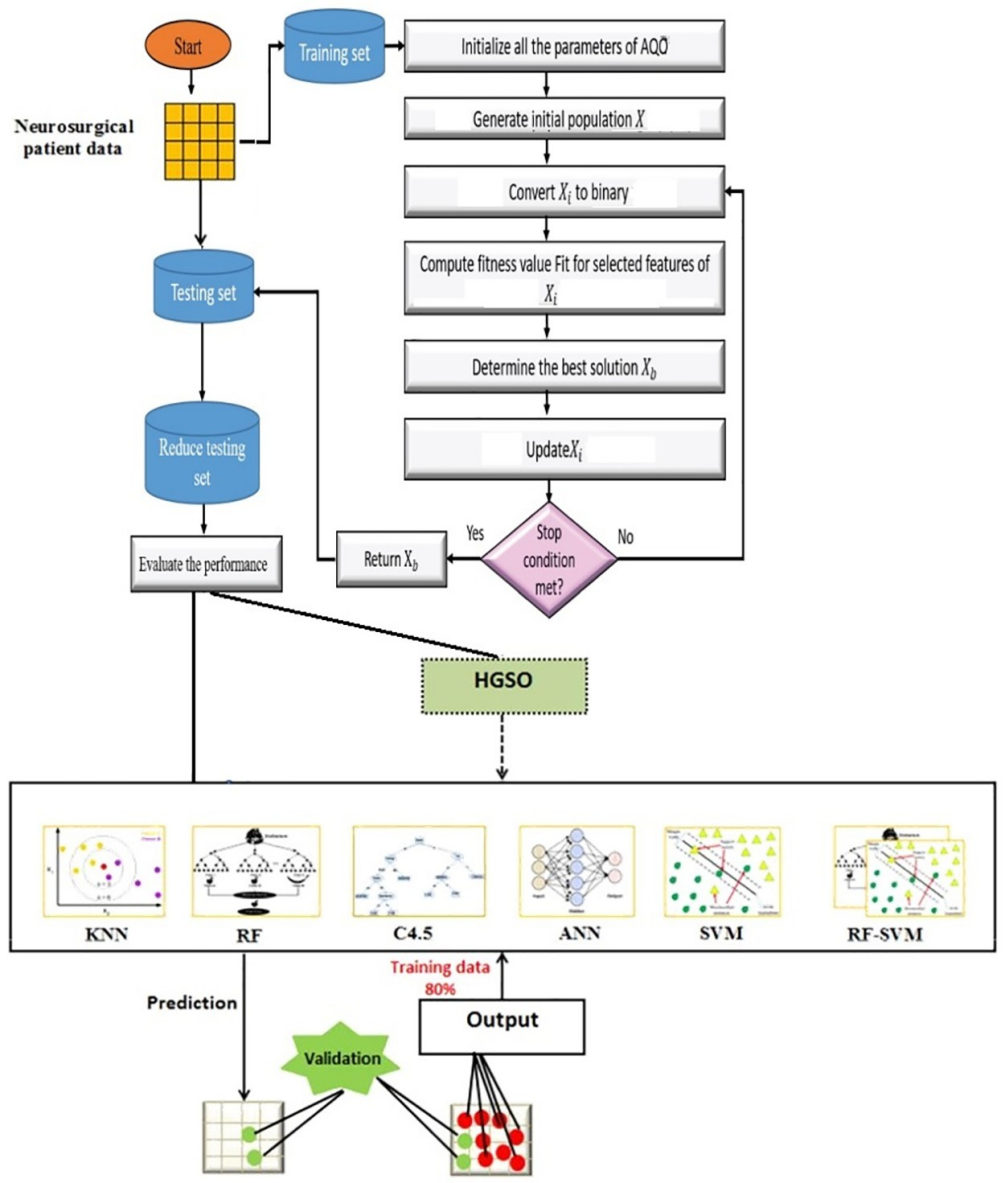
Framework of the machine learning models trained and evaluated based one AQO with and without using HGSO to classify patient status outcome.

The goal of the present study is to provide an efficient feature selection model using ML techniques based on AQO optimized HGSO framework for developing a Neurosurgical prediction model. Several ML techniques were examined. These techniques were investigated as a standalone without the use of HGSO then compared to its counterparts after applying HGSO. To determine the best performing technique that can be used in the prediction model, the framework shown in (Fig 2) is developed.

Neurosurgical patient data set contains 3000 records of patients. The dataset is balanced containing 800 records of patient having “mortality” outcome status, 700 records have “morbidity” outcome status, 850 records have “the same” outcome status, and 650 records have “improved” outcome status. The preprocessing activities were performed. To predict the status outcome of Neurosurgical patients with high accuracy, after data set processing we have 2 cases.

- **Case 1**: K-NN, RF, C4.5, ANN, SVM, RF-SVM algorithms were applied and their performance is evaluated.
- **Case 2**: feature selection technique (HGSO) is applied in conjunction with the algorithms stated in Case 1 above.

### 4.2 Experiments

The techniques (K-NN, RF, C4.5, ANN, SVM, RF-SVM) were tested on the neurosurgical dataset that is developed in this work. The proposed RF-SVM based on HGSO algorithm was utilized. The evaluation metrics for (K-NN, RF, C4.5, ANN, and SVM) were tested with different pop sizes (from 25 to 50) with and without using AQO-based HGSO classifiers were calculated. The results indicate that the double machine learning algorithm (RF-SVM) based on HGSO algorithm can perform well in practice under (pop size = 30). Here the measurements of the proposed algorithm are presented.

### 4.3 Evaluation metrics

This study was implemented using Python platform and a comparative analysis was performed. The Recall, Precision, F-measure, Accuracy, and Sensitivity were defined as follows [54, 55]:

$$\begin{array}{c} Accuracy=\frac{TP+TN}{TP+TN+FP+FN} \end{array}$$ (20)

$$\begin{array}{c} Recall=\frac{TP}{TP+FN} \end{array}$$ (21)

$$\begin{array}{c} Precision=\frac{TP}{TP+FP} \end{array}$$ (22)

$$\begin{array}{c} F-measure=2\times \frac{Precision*recall}{Precision+recall} \end{array}$$ (23)

where *TP*, *FP*, *FN* and *TN* represent True Positive, False Positive, False Negative, and True Negative respectively.

## 5 Results and comparative analysis

**Fig 3:**
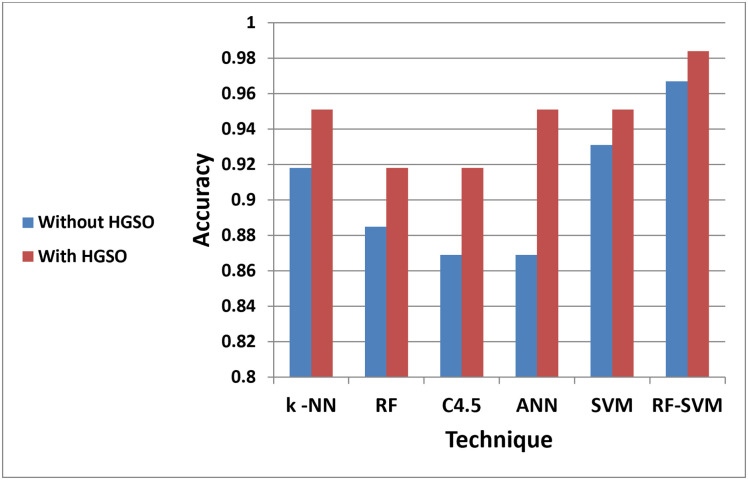
The accuracy for (KNN, RF, C4.5, ANN, SVM and RF-SVM) classifiers with and without using HGSO.

In this section, a comparison between the accuracy of the proposed model with and without applying feature selection technique is introduced. The comparative results of different machine learning models are performed. It is found that the classification process affected by several attribute values in the data. the importance of the features is explored by finding the accuracy of the dataset. Five single classifiers (K-NN, RF, C4.5, ANN, and SVM) and one double classifier (RF-SVM) were applied to the dataset. Then, reapplied again while based on HGSO to determine if there is any enhancement in the prediction by implementing HGSO. The best attributes for prediction were determined by the selected threshold value and the accuracy of the different classifiers as summarized in Table 2 and illustrated in (Fig 3).

**Table 2:** The accuracy for (KNN, RF, C4.5, ANN, SVM and RF-SVM) classifiers based on HGSO.

KNN	RF	C4.5	ANN	SVM	RF-SVM
0.962	0.926	0.924	0.959	0.961	0.992

RF-SVM based on HGSO showed better results when compared with other classifiers. The run parameters are shown in Table 3 while the results are shown in Table 4.

**Table 3:** Run parameters.

# runs	Domain Range	Log	Epoch	Pop Size	Lsa Epoch
100	[-1,1]	False	50	30	100

**Table 4:** Comparison of classification model results.

Model	Evaluation Measure	Without HGSO	HGSO
K-NN	Recall	0.944	0.981
	Precision	0.962	0.966
	F-Measure	0.953	0.972
	Accuracy	0.923	0.962
RF	Recall	0.912	0.947
	Precision	0.963	0.967
	F-Measure	0.937	0.956
	Accuracy	0.861	0.926
C4.5	Recall	0.893	0.947
	Precision	0.952	0.967
	F-Measure	0.926	0.956
	Accuracy	0.891	0.924
ANN	Recall	0.893	0.966
	Precision	0.962	0.980
	F-Measure	0.926	0.974
	Accuracy	0.899	0.959
SVM	Recall	0.965	0.982
	Precision	0.962	0.984
	F-Measure	0.965	0.979
	Accuracy	0.947	0.961
RF-SVM	Recall	0.983	0.987
	Precision	0.983	0.986
	F-Measure	0.983	0.993
	Accuracy	0.976	0.992

**Fig 4:**
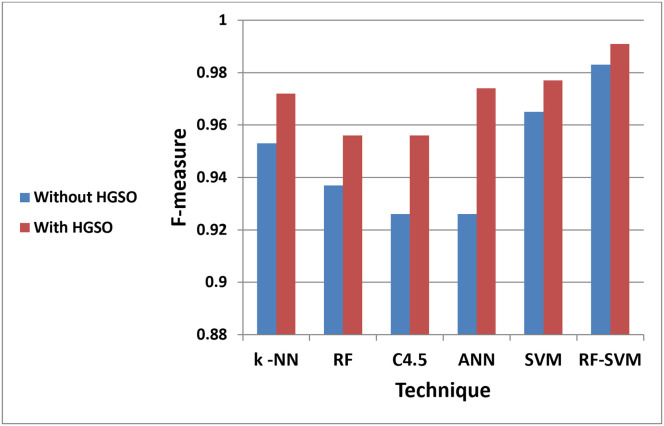
RF-SVM has the largest values of F-measure.

**Fig 5:**
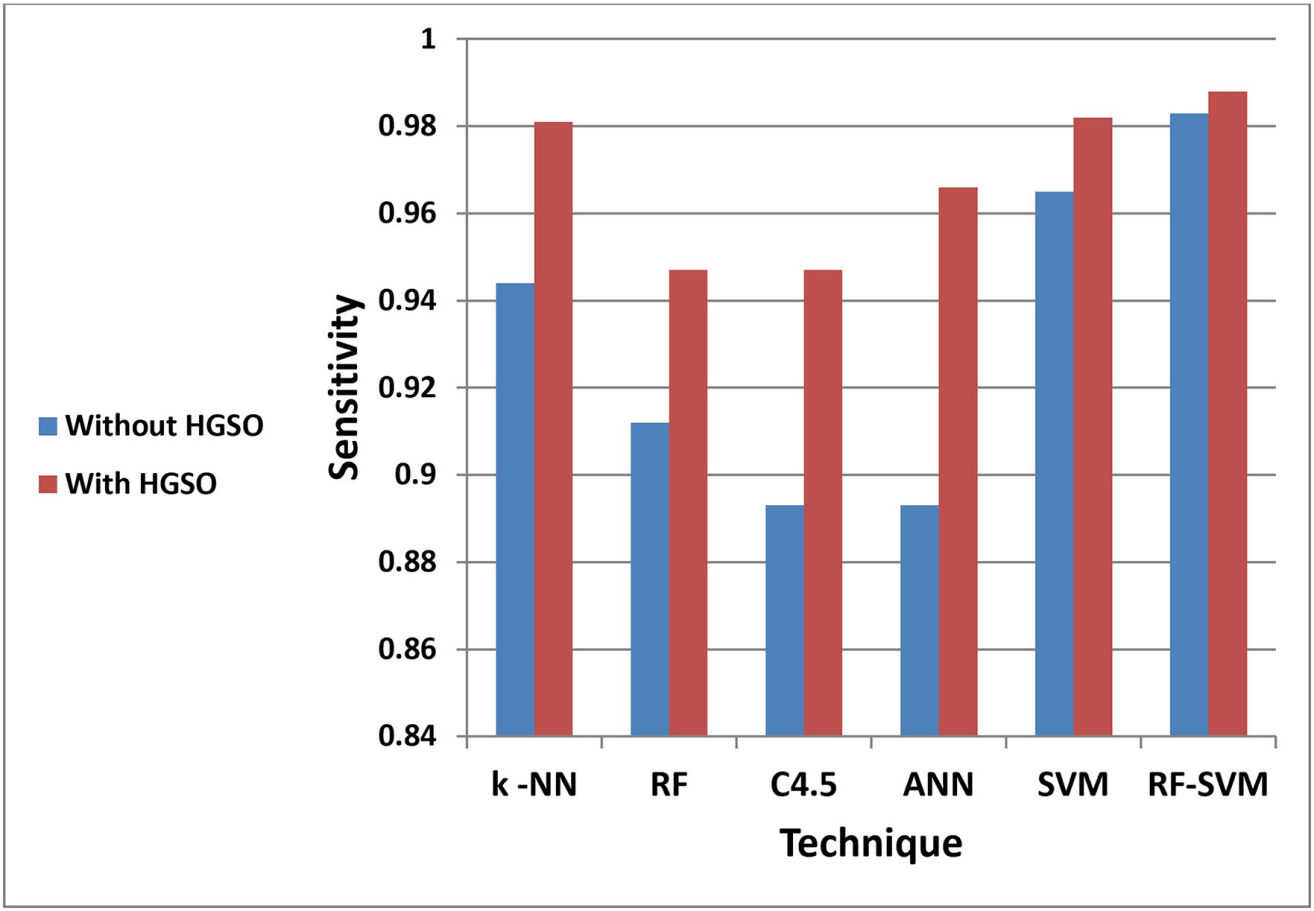
RF-SVM based on AQO HGSO sensitivity.

The chart in (Fig 4) highlights the differences in the F-measure while the sensitivity is shown in (Fig 5). It clearly shows the importance of feature selection based on AQO HGSO in the generated model.

The comparison between the 6 classifiers is recorded in Table 5 based on feature selection model HGSO according to the average recall, precision, F-measure, accuracy and sensitivity. As in Table 5 RF-SVM using 32 features had the smallest SD value among the tested methods and the highest f-measure and accuracy, respectively.

**Table 5:** Results of different parameters of ML classification models.

Model	Measure	HGSO	# Features	Min	Max	SD
K-NN	Recall	0.981	30	0.94	0.99	0.025
	Precision	0.966		0.92	0.98	0.03
	F-Measure	0.972		0.93	0.99	0.03
	Accuracy	0.962		0.88	0.97	0.045
RF	Recall	0.947	30	0.87	0.96	0.045
	Precision	0.967		0.93	0.98	0.025
	F-Measure	0.956		0.92	0.96	0.02
	Accuracy	0.926		0.91	0.95	0.005
C4.5	Recall	0.947	30	0.88	0.95	0.035
	Precision	0.967		0.95	0.97	0.01
	F-Measure	0.956		0.9	0.96	0.03
	Accuracy	0.924		0.84	0.93	0.045
ANN	Recall	0.966	30	0.87	0.95	0.04
	Precision	0.980		0.94	0.96	0.03
	F-Measure	0.974		0.990	0.99	0.045
	Accuracy	0.959		0.83	0.99	0.08
SVM	Recall	0.982	30	0.95	0.96	0.025
	Precision	0.984		0.95	0.99	0.02
	F-Measure	0.979		0.93	0.99	0.03
	Accuracy	0.961		0.93	0.97	0.02
RF-SVM	Recall	0.987	30	0.96	0.97	0.02
	Precision	0.986		0.95	0.96	0.025
	F-Measure	0.993		0.96	0.97	0.02
	Accuracy	0.992		0.96	0.98	0.02

**Fig 6:**
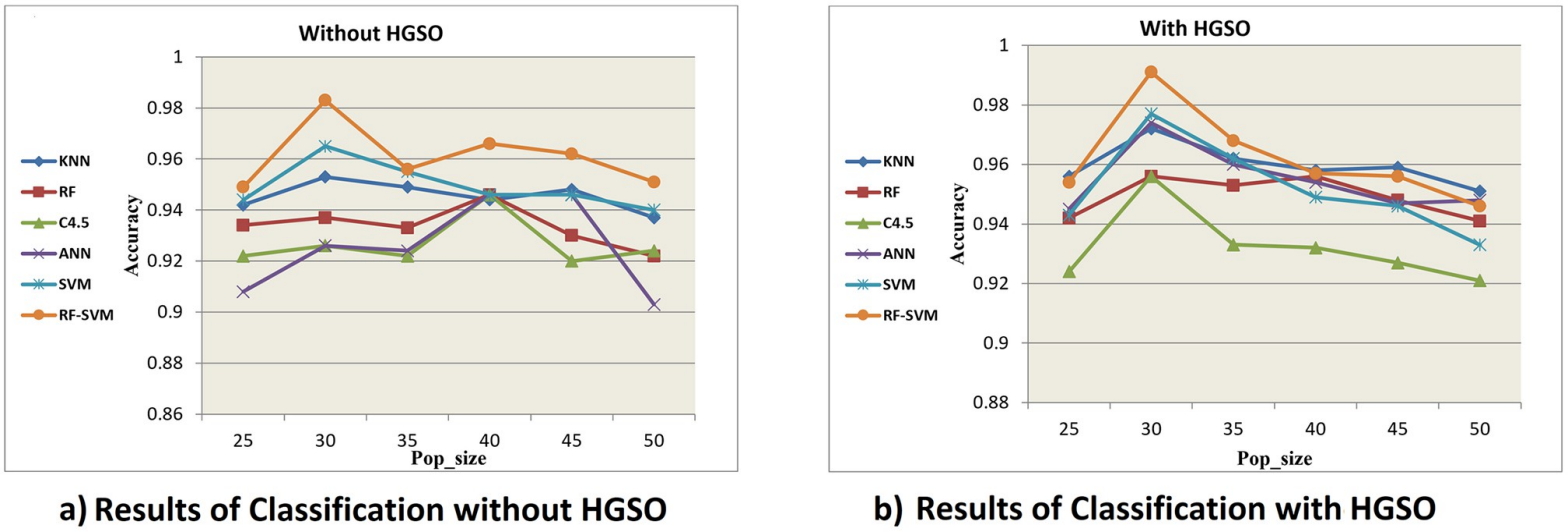
Classification results for different number of *pop*_*size* (25–50) for each classifier employed: KNN, RF, C4.5, ANN, SVM, RF-SVM, without HGSO (a) and with HGSO (b). (a) Results of Classification without HGSO. (b) Results of Classification with HGSO.

As shown in (Fig 6), results indicate that RF-SVM is the best classification algorithm with an accuracy of 99.2% (at pop size = 30).

Table 6 shows the average overall F-measure results of *pop*_*size*= 25, 30, 35, 40, 45, and 50 for K-NN, RF, C4.5, SVM, and RF-SVM both with and without HGSO model. As shown in Table 6, *pop*_*size* = 30 outperformed at all results, and the average overall F-measure for RF-SVM with AQO HGSO achieved 99.3%. It scored the best results.

**Table 6:** Comparative analysis of machine learning models with different pop sizes.

	Pop Size						
	Model	25	30	35	40	45	50
Without HGSO	K-NN	0.948	**0.923**	0.954	0.949	0.952	0.941
	RF	0.937	**0.861**	0.938	0.951	0.934	0.927
	C4.5	0.926	**0.891**	0.928	0.953	0.925	0.929
	ANN	0.911	**0.899**	0.929	0.952	0.951	0.909
	SVM	0.950	**0.947**	0.959	0.952	0.951	0.945
	RF-SVM	0.957	**0.976**	0.961	0.971	0.966	0.956
With HGSO	K-NN	0.966	**0.962**	0.976	0.964	0.964	0.958
	RF	0.954	**0.926**	0.964	0.968	0.956	0.947
	C4.5	0.934	**0.924**	0.945	0.948	0.936	0.926
	ANN	0.955	**0.959**	0.969	0.969	0.954	0.951
	SVM	0.953	**0.961**	0.968	0.955	0.955	0.938
	RF-SVM	0.961	**0.992**	0.973	0.962	0.967	0.953

## 6 Statistical tests

The statistical test analysis was conducted using Wilcoxon’s test based on accuracy metric. The Wilcoxon test is a non-parametric test [56], therefore it has less assumptions than parametric tests such as t-test. As a result, the Wilcoxon test is performed when the t-test for dependent samples fails to meet its boundary criteria.

**Fig 7:**
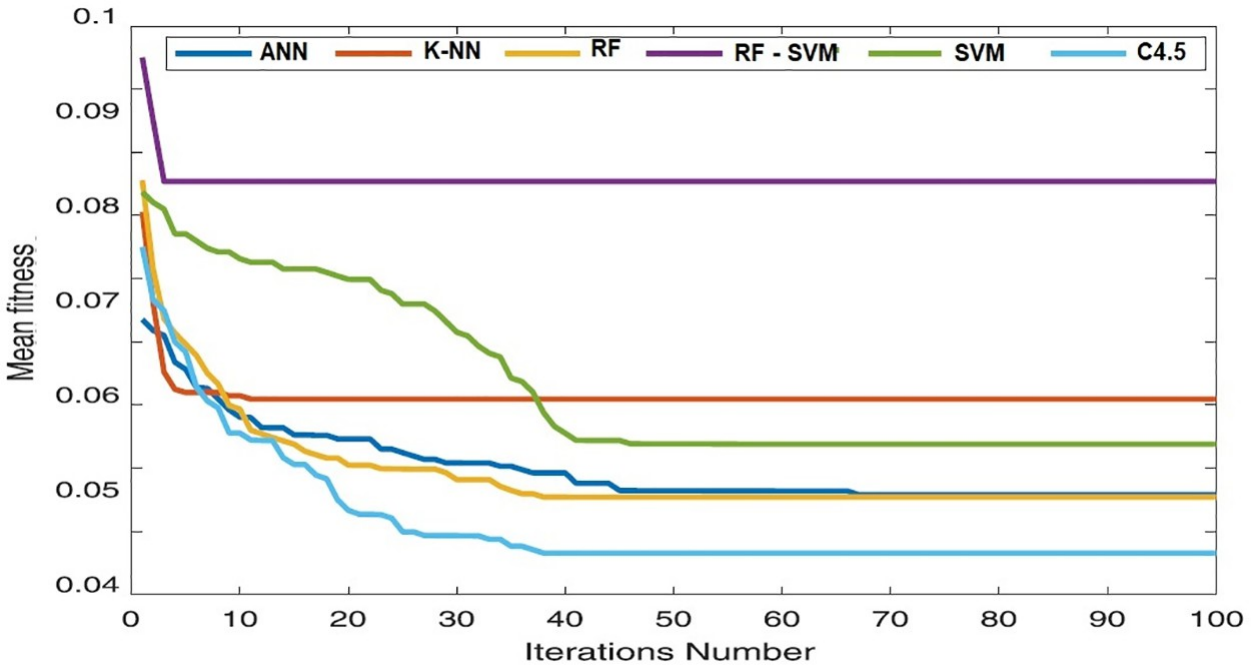
Convergence curve of used algorithms with HGSO over the selected dataset.

**Fig 8:**
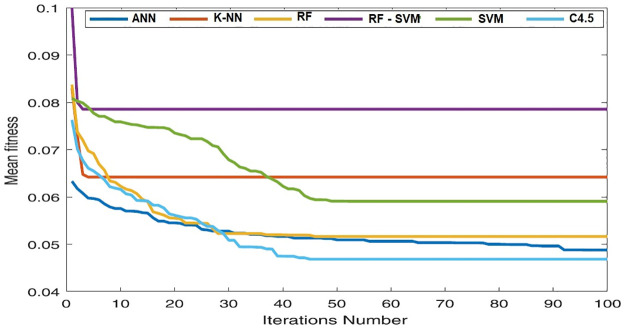
Convergence curve of used algorithms without HGSO over the selected dataset.

Wilcoxon test may be computed using the difference between the two dependent values. The absolute value of the difference is utilized to determine the rankings once the difference is computed. It is crucial to keep in mind the first indication of discrepancies. All experiments were designed to be run 30 times with 10 solutions and 100 max iterations. (Fig 7), and (Fig 8) compare the accuracy performance and the number of the selected features for ANN, K-NN, RF, RF-SVM, SVM and C4.5 algorithms with and without HGSO over the selected dataset.

## 7 Conclusions

Recently, machine learning is being viewed as the most important field for the classification of large datasets particularly in medical domain. Its techniques improve the capability of human in treating large datasets by finding the important attributes in the dataset. This study explores the importance of RF-SVM based on HGSO by performing different measurements in a Neurosurgical dataset. The Recall, Precision, F-measure, Accuracy and Sensitivity of K-NN, RF, C4.5, ANN, SVM, and RF-SVM classifiers were recorded and compared. The accuracy of the classifiers ranged from 92.4% to 99.2%. The RF-SVM based on HGSO model produced the highest accuracy and showed better results when compared with other classifiers.

Machine learning remains in the forefront of future studies in healthcare applications. It can be used to identify and diagnose diseases based on ML ability to classify data. This not only reduces the length of the diagnosis process but also reduces mistakes made by doctors. As medical training takes a long time. The methodology applied here can be used for medical imaging diagnosis which is promising where combination of data from multiple data sources can lead to a different progression. Moreover, it will be interesting to implement the algorithm on crowdsourcing data collection and analysis. Finally, there are various domains for ML application in healthcare.

## Informed consent

Informed consent was from the subjects involved in the study where applies. The data data supplied by the hospital was anonymized.
